# Predictors of Productivity and Leisure for People Aging with Intellectual Disability

**DOI:** 10.1177/00084174211073257

**Published:** 2022-01-17

**Authors:** Eilish King, Joan Brangan, Mary McCarron, Philip McCallion, Fathima Rosmin Bavussantakath, Mary-Ann O’Donovan

**Keywords:** Intellectual disability, Leisure activities, Occupational therapy, Older adult, Work, Activités de loisir, déficience intellectuelle, ergothérapie, personnes âgées, travail

## Abstract

**Background.** Adults aging with intellectual disability (ID) face barriers to engagement in occupation. Greater understanding of factors that affect engagement in work and leisure occupations is required to support occupational engagement in this population. **Purpose.** Identify predictors of engagement in work and leisure occupations for adults aging with an ID, and consider implications for occupational therapy practice. **Method.** Data from wave 2 of the Intellectual Disability Supplement to the Irish Longitudinal Study on Aging (IDS-TILDA) was analyzed using regression analysis to identify predictors of engagement in work and leisure occupations for adults aging with an ID. **Findings.** Adults who had difficulty getting around their home environment, poor physical health, or older age were less likely to engage in work and leisure activities. **Implications.** Occupational therapists can support adults aging with ID to age in place. Occupation-focused health promotion could enhance well-being through engagement in occupation.

## Résumé

**Description.** En vieillissant, les personnes qui ont une déficience intellectuelle font face à des obstacles sur le plan de l’engagement occupationnel. Une meilleure compréhension des facteurs qui affectent l’engagement dans les activités de travail et de loisir est donc nécessaire. **But.** Déterminer les prédicteurs de l’engagement dans les activités de travail et de loisir lors du vieillissement chez les personnes qui présentent une déficience intellectuelle, et envisager les conséquences pour l’ergothérapie. **Méthodologie.** Les données de la deuxième vague de l’étude *Intellectual Disability Supplement to the Irish Longitudinal Study on Aging* (IDS-TILDA) ont fait l’objet d’une analyse de régression pour y trouver les prédicteurs de l’engagement dans les activités de travail et de loisir lors du vieillissement chez les personnes présentant une déficience intellectuelle. **Résultats.** Les adultes qui avaient du mal à se déplacer dans leur environnement habituel, qui étaient en mauvaise santé physique ou qui étaient plus âgés étaient moins susceptibles de participer à des activités de travail et de loisir. **Conséquences.** Les ergothérapeutes peuvent aider les adultes qui présentent une déficience intellectuelle à vieillir dans leur milieu. Des activités de promotion de la santé axées sur l’engagement dans les activités peuvent améliorer leur bien-être.

## Introduction

The population of people aging with an intellectual disability (ID) is growing in Ireland and internationally (Ouellette-Kuntz et al., 2016). In Ireland, there were a total of 28,388 people registered on the National Intellectual Disability Database (NIDD) in 2017, representing a prevalence of 5.96 per 1,000 people (Hourigan et al., 2017). Of these, 11,789 registrants were aged 35 years or older, representing 41.9% of those registered on the NIDD, demonstrating that Ireland has a significant population of people aging with an ID (Hourigan et al., 2017). The database also shows that approximately 31% of people with ID registered were availing of occupational therapy services in 2017 (Hourigan et al., 2017).

There is growing international evidence describing occupational therapy practice with people with ID. Blaskowitz et al. (2021) and Johnson et al. (2019) outlined a broad scope of occupational therapy practice with adults with intellectual disabilities, including training, prompting, activities of daily living (ADL), health management, community participation programs, vocational training, advocacy, building social capital, environmental modification, and assistive technology. These studies examined adults aged 18 years and older with an ID, and focused on identifying current and future areas for occupational therapy practice development. Other scoping reviews have explored the role of environmental supports in the lives of people aging with ID (Washington et al., 2021). Mahoney et al. (2016) examined the occupational engagement of adults with ID participating in a day program in the United States. Haines et al. (2018) described the collaboration between occupational therapists and direct support staff to promote opportunities for people with profound intellectual and multiple disabilities to engage in a variety of meaningful occupations. Hynes and Harb (2017) outlined occupational therapy involvement in working with adults with intellectual disabilities who wish to access supported employment services to occupational therapists.

Occupational therapists are primarily concerned with promoting health and well-being through occupation, and can work with adults with ID across the lifespan (Johnson et al., 2019). Fesko et al. (2012) noted that maintaining an active routine with engagement in meaningful activities promotes positive mental health, higher self-esteem, and self-determination for adults with ID.

Johnson et al. (2019) reported that occupational therapists are ideally placed to work with people with ID to support occupational engagement, but that the occupational therapy profession is not currently addressing the occupational needs of this population in a satisfactory manner. Mahoney et al. (2019) found that many occupational therapists working with older adults aging with an ID felt they would benefit from additional training and education to meet the needs of this growing population. Thus, understanding how older adults with ID wish to engage and the factors that need to be in place at the individual, environmental, and cultural levels can inform such training.

Washington et al. (2021) concluded that further research regarding environmental supports for adults aging with ID was warranted, utilizing occupational therapy perspectives and conceptual models. Consideration of the needs of adults aging with ID is important, as this population may require increased levels of support for daily activities and occupations as they grow older, (Kåhlin et al., 2016; King et al., 2017). Occupational therapists have a diverse and important role to play in enabling adults aging with ID to engage in meaningful occupation (Johnson et al., 2019; King et al., 2017). Previous research using Intellectual Disability Supplement to the Irish Longitudinal Study on Aging (IDS-TILDA) data explored participation in work activities from a social participation perspective (McCausland et al., 2019). It is important that occupational therapists understand the particular environmental, personal, or occupational factors that can affect participation in this growing population. The present study seeks to contribute to this, with an occupational therapy perspective applied to a large quantitative dataset that is nationally representative, for the first time and thus affording the opportunity to understand the data in a way not previously explored. This paper will focus on work and leisure as two important domains of daily occupations for adults aging with ID.

### Work or Productivity

Occupational therapists take a broad perspective of work or productivity (hereafter referred to productivity in this paper), which includes any meaningful productive role that contributes to the community or society (Townsend, 2002). A growing body of literature from the IDS-TILDA and other studies recommend that people with ID are a diverse group, so a broad perspective of participation in work-related activities should be taken when considering adults aging with ID (Lysaght et al., 2017; McCausland et al., 2019). This could include participation in day services, training, volunteering, or other meaningful productivity occupations, as well as paid employment (McCausland et al., 2019). This is especially relevant for adults aging with ID, who may experience health-related changes, or broader changes within the physical, social or cultural environment.

Presently in Ireland, demand is increasing for all types of work and productivity options, including supported employment, further education/training, or traditional day services (Hourigan et al., 2017). Day services can provide important opportunities for forming and maintaining relationships and engagement in activities, including physically oriented, artistic and creative activities, as well as providing a meaningful social role (Campbell, 2012; McGlinchey et al., 2013).

Inclusive education initiatives for people with ID also continue to grow and develop within Ireland and internationally (O’Brien et al., 2018). The benefits of such programs are numerous, with positive outcomes for employment, and self-determination, with higher ratings of self-reported good health, employment, independent living skills, and social connections (Ross et al., 2013).

Employment rates of people with ID in Ireland remain low. McCausland et al. (2019) reported a rate of less than 10% of the IDS-TILDA population was in paid employment. Using IDS-TILDA data, McCausland et al. (2019) found the majority of the population is engaged in some form of productive occupation, such as a day service or training initiative. Higher levels of social contact and friends were associated with greater participation in productivity roles, such as training, day service, or employment in their study.

### Leisure

Leisure, or activities undertaken for enjoyment, which may support positive aging outcomes, are an important component of community involvement, and an opportunity to experience autonomy for people with ID (Charnley et al., 2019; Mihaila et al., 2020). Adults aging with ID have identified that engagement in leisure occupations is important, and continue to engage in social leisure activities, but the location in which they participate in leisure may change over time (McCarron et al., 2017; Schepens et al., 2019). People with ID may experience barriers to accessing meaningful leisure occupations. Charnley et al. (2019) reported that the concerns of disability services staff regarding risk, and availability of staff support, served as barriers to participation in leisure for adults with intellectual disabilities. Other studies found barriers to leisure participation may include transport, limited financial resources to spend on leisure occupations, older age, availability of accessible physical facilities, and fear of stigma or unwelcoming environments for adults with ID (Charnley et al., 2019; Dusseljee et al., 2011; Melbøe & Ytterhus, 2017).

### Rationale, Study Aims, and Research Question

Recent scoping reviews highlighted current and future areas for the occupational therapy practice with adults with ID. This study adds to this work by examining the needs of older adults with ID, and focusing on predictors of engagement in work and leisure activities.

Previous research using IDS-TILDA data examined participation in ADL (King et al., 2017). This study aims to examine factors that predict preclusion from engagement in productivity and leisure occupations, as these are also important domains of daily occupation. This study extends previous research using IDS-TILDA data conducted by McCausland et al. (2019). This paper examines productivity and leisure occupations from an occupational therapy perspective to build an understanding of these important domains of daily occupation for adults aging with ID. In this way, the present study has particular relevance for occupational therapists working with adults aging with ID.

A growing number of qualitative studies have provided valuable insights into the occupational engagement of adults with ID. It is also important to understand the barriers to participation that may be experienced across a large cohort of adults aging with ID in Ireland. This study examines predictors of engagement in work and leisure occupations for a nationally representative sample of adults aging with ID in Ireland from an occupational therapy perspective.

Existing non occupational therapy-based research often uses demographic factors such as age, sex, level of education, geographic location, as potential factors influencing participation in work or leisure activities, for example,. (Dusseljee et al., 2011; McCausland et al., 2019; McGlinchey et al., 2013). The present study examined selected objective factors related to the person, environment, and occupation as “key indicators,” or potential factors that influence participation in leisure and work occupations.

The aim of this study was to address the gap in current research by seeking answers to the following question: What are the predictors of preclusion from engagement in productivity and leisure activities for adults aging with an ID in Ireland? In response, the study aimed to recommend strategies to enable people with ID to continue to participate in meaningful productivity and leisure occupations as they grow older, which would be of particular interest to occupational therapists working with this population.

## Methods

### IDS-TILDA

This study used data from wave 2 of the IDS-TILDA. The data were collected in 2014. IDS-TILDA is a prospective longitudinal study concerned with aging with an ID and collects data in 3-year cycles. Since 2014, there have been additional waves of data collection in IDS-TILDA. The researchers had access to wave 2 data. This data are relevant as it provides important information about the population of adults aging with an ID in Ireland, and examines factors related to the person, environment, and occupation to inform implications for occupational therapy practice.

IDS-TILDA gathers data across a wide variety of domains related to health, well-being, and quality of life of adults aging with ID in Ireland. The IDS-TILDA sample was drawn from the NIDD, an administrative service planning database that gathers information on people with ID accessing specialized health services in Ireland (Hourigan et al., 2017).

Data were collected using computer-assisted personal interviews (CAPI) by the research team including some of the authors. CAPI was used in structured interviews, which were completed by respondents who self-reported, either alone or in collaboration with a support person, and participants who completed the questionnaire by proxy only. Proxy respondents were required to have known the person for at least 6 months prior to interview, as the selection of proxy respondents with a good level of knowledge of the person has been found to increase the accuracy of responses for people with ID (Foran et al., 2013).

The IDS-TILDA includes people aged 40 years and older with all levels of ID from a range of living situations. The IDS-TILDA wave 1 sample consisted of 753 persons in 2010. There was a 94% retention rate between wave 1 and wave 2 of IDS-TILDA, giving a population of 708 participants in wave 2 (Burke et al., 2014). When accounting for missing data, this resulted in a total population of 699 participants included in data analysis.

IDS TILDA wave 2 data were analyzed using Statistical Package for Social Sciences (SPSS Version 21.0). A cut-off point of *p* < .05 was used to establish statistical significance in all analyses.

### Ethical Considerations

IDS TILDA has received ethical approval from the Trinity College Faculty of Health Sciences Ethics Committee, and from the 138 ID service providers involved in IDS-TILDA for both wave 1 and wave 2 (Burke et al., 2014). IDS-TILDA has a comprehensive data protection protocol to ensure confidentiality for participants.

Informed consent was obtained from all participants. Consent was viewed as an ongoing process (Dewing, 2007). A minimum 7-day period elapsed between receipt of accessible study information with consent forms, and follow-up by the researcher to allow sufficient time to consider study participation. Researchers reaffirmed consent with participants at the beginning, and during the interview. Participants were offered the opportunity to split their interviews over multiple visits, to take breaks, and to stop or withdraw at any time.

### Study Procedures and Data Analysis

Descriptive statistics were completed to build the demographic profile of IDS-TILDA participants. To ensure representation of a broad range of adults aging with ID, variables that included participants who completed the IDS-TILDA with support, or by proxy were selected. Variables were selected on the basis of relevance to broad occupational therapy concepts of “person,” “environment”, and “occupation” from the IDS-TILDA dataset. However, subjective factors such as spirituality, or the influence of the cultural or institutional environment could not be accurately gathered in this manner, and were omitted from the analysis. A summary of variables used in the analysis is provided in Table 1.

**Table 1:** IDS-TILDA Variables Selected for Analysis

IDS-TILDA variable and response options	
Self-rated physical health	(a) Good self-rated physical health (b) Poor self-rated physical health
Level of ID	(a) Mild level of ID (b) Moderate level of ID (c) Severe/profound level of ID
Self-rated mental health	(a) Good self-rated mental health (b) Poor self-rated mental health
Level of difficulty mobilizing around home environment or community	(a) Difficulty mobilizing around home environment (b) No difficulty mobilizing around home environment (c) Difficulty traveling around local community, or does not travel around local community (d) No difficulty traveling around local community
Living situation	(a) Independent/with family (b) Community group home (c) Residential center
Presence of friends outside the home	(a) Friends outside the home (b) No friends outside the home
Support required for activities of daily living (ADL) or Instrumental activities of daily living (IADL)	(a) Support required for **either** ADL or IADL (b) Support required for **both** ADL and IADL

*Note:* ID = intellectual disability; IDS-TILDA = Intellectual Disability Supplement to the Irish Longitudinal Study on Aging.

#### Statistical applications: Productivity variable analysis

A productivity variable was constructed from the dataset based on participation in employment, sheltered workshops, day services, voluntary work, education, or looking after home and family. Day services were included in this group, as adults with ID have reported that they perceive participation in day services to be a meaningful productivity role (McGlinchey et al., 2013). The variable categorized participants as either engaged in employment, sheltered workshops, day services, volunteering, education, or looking after home and family (known as “engaged in a productivity role”), or unemployed retired, or unable to work due to illness (known as “not currently engaged in a productivity role”).

Cross tabulations and chi-square tests of independence were completed in an initial examination of the association between IDS-TILDA demographic information, self-reported health, presence of friends outside the home, difficulty mobilizing around home and local community, support for ADL, and the productivity variable.

Variables that were significant in chi-square tests for independence were included in binary logistic regression models for productivity variables.

#### Statistical applications: Leisure variable analysis

The leisure variable was constructed from responses to frequency of participation in specific leisure activities (e.g., daily, weekly, monthly, or less frequently). The mean value was 26.6, with a *SD* of 13.5. Leisure activities included going for coffee/tea, attending or participating in sports events, engaging in arts groups, cinema, hobbies, visiting family or friends, going to the library, shops, or social clubs, as well as “other” leisure activities. The responses were reverse coded, then summed to create a continuous leisure variable with consideration to both the number and frequency of leisure activities that people engaged in. Those who engaged in a wider variety of leisure activities on a more regular basis received higher leisure engagement scores than those who engage in less leisure activities on a less regular basis.

The Kolmogorov–Smirnov test was completed on the continuous leisure variable, with a significant value of .064 indicating normality of distribution of values, supporting the value of multiple linear regression.

Independent samples *t*-tests and one way between groups analysis of variance (ANOVA) were completed to identify factors that significantly affect participation in leisure activities. Factors that were identified as significant in the independent samples *t*-tests, and one-way ANOVA were included in multiple linear regression.

## Findings

Demographic information on the IDS-TILDA wave 2 population is outlined in Table 2. The majority of IDS-TILDA participants were aged between 50 and 64 years (51.1%), and resided in community group homes (43.6%), or residential centers (40.3%). 55.8% of the IDS-TILDA participants were female, and the majority of IDS-TILDA participants were single (98.6%).

**Table 2:** Demographic Profile of IDS-TILDA Wave 2

	Frequency (n= 699) (n = 8 cases excluded from overall analysis due to incomplete data)	%
Age (n = 47 missing):		
44–49 years	187	28.7
50–64 years	333	51.1
65 + years	132	20.2
Sex:		
Male	313	44.2
Female	386	55.8
Marital status (n = 50 missing):		
Single	640	98.6
Other, for example, Married	9	1.4
Level of ID (n= 54 missing):		
Mild	153	23.7
Moderate	300	46.5
Severe/Profound	192	29.8
Living Situation		
Independent/Family	112	16
Community group home	305	43.6
Residential center	282	40.3

*Note:* ID = intellectual disability; IDS-TILDA = Intellectual Disability Supplement to the Irish Longitudinal Study on Aging.

Occasionally, missing data can occur within the data as some questions were posed to self-reporting respondents, or participants did not wish to answer certain questions. This was managed using pairwise deletion, so that other responses from the participants could be included elsewhere in the dataset.

### Productivity

The majority (85.4%, n = 597) of respondents reported to be engaged in a work-related, or productive role. Participants who reported to be engaged in a productivity role tended to report higher percentages of having friends outside of their homes (90.1%), and no difficulty in moving around their homes. The majority of younger participants (90.4% of those aged 40–49 years) were engaged in a productivity role, and the majority of those living independently or with their families (96.5%) reported to be engaged in a productivity role.

Table 3 shows the results of cross tabulation and chi-square tests for engagement in productivity activities. Results of the cross tabulations are presented horizontally in the table.

**Table 3:** Cross Tabulation and Chi-Square Results for Engagement in Productivity Activities

Productivity:	Engaged in productivity role (n = 597, 85.4%)	%	Not engaged in productivity role (n = 102, 14.6%)	%	Chi-square	*P*
Sex:					0	>.999
Female	335	85.5	57	14.5		
Male	262	85.3	45	14.7		
Age:					26.4	*<.001
40–49 years	179	90.4	19	9.6		
50–64 years	311	88.1	42	11.9		
65 + years	107	72.3	41	27.7		
Self-rated physical health (n = 8 missing):					35.5	*<.001
Good	517	89.1	63	10.9		
Poor	72	66.7	36	33.3		
Self-rated mental health (n = 14 missing):					13.2	*<.001
Good	446	88.8	56	11.2		
Poor	137	77.4	40	22.6		
Level of ID (n = 50 missing):					7.3	*.03
Mild	135	88.2	18	11.8		
Moderate	261	87	39	13		
Severe/Profound	151	78.6	40	20.8		
Physical Environment (Home) (n = 3 missing):					52.9	*<.001
No difficulty navigating home environment	518	90.1	57	9.9		
Difficulty navigating home environment	76	63.9	43	36.1		
Physical environment (Community) (n = 7 missing):					28.9	*<.001
No difficulty accessing local community	236	95.2	12	4.8		
Difficulty/does not access local community	354	79.7	90	20.3		
Social environment (n = 5 missing):					14.7	*<.001
Friends outside home	353	90.1	39	9.9		
No friends outside home	239	79.4	62	20.6		
Living situation					16.4	*<.001
Independent/Family	109	96.5	4	3.5		
Community group home	260	85.8	43	14.2		
Residential center	228	80.6	55	19.4		
Support for activities of daily living (n = 24 missing):					6.9	*.009
Support for either ADL or IADL	97	94.2	6	5.8		
Support for both ADL and IADL	476	83.7	93	16.3		

*Note:* ADL = activities of daily living; ID = intellectual disability; IADL = instrumental activities of daily living.

*Indicates significance, *p* < .05.

Age, self-rated physical health, self-rated mental health, level of ID, difficulty navigating the home environment, and accessing the local community, presence of friends outside the home, type of living situation, support required for ADL were all identified as significant factors of engagement in chi-square test (*p* < .05). All significant factors were included in logistic regression analysis. Sex was not identified as a significant factor.

#### Regression

Age, level of ID, living situation, physical health, mental health, physical environment (home and community), social environment, and support required for ADL were entered into a binary logistic regression. Sex was also included in the regression as a demographic variable. Results of binary logistic regression with adjusted odds ratio are shown in Table 4. The full model containing all 10 independent variables was statistically significant, (n = 593, *p* < .001) and the model explained between 14.3% (Cox & Snell R Square) and 25.2% (Nagelkerke R square) of variance in engagement in a productivity role, and classified 86.2% of cases correctly.

**Table 4:** Binary Logistic Regression with Adjusted Odds Ratio for Predictors of Productivity

Engaged in productivity role (n = 597, 85.4%)	*P*-value	Adjusted odds ratio [95% confidence interval]
Age:		
Age 40–49 years (Ref. group)	*.006	
Age 50–64 years	.585	1.2 [.62, 2.32]
Age 65 + years	*.005	2.79 [1.36, 5.72]
Sex:		
Female	.723	.91 [.54, 1.53]
Level of ID:		
Level of ID: Mild (Ref group)	.257	
Level of ID: Moderate	.181	.6 [.28, 1.27]
Level of ID: Severe/profound	.763	.88 [.39, 1.99]
Living situation:		
Independent/with family (Ref group)	.293	
Community group home	.176	2.39 [.68, 8.44]
Residential setting	.368	1.81 [.5, 6.56]
Physical health:		
Physical health: Poor	*.04	1.89 [1.03, 3.46]
Mental health:		
Mental health: Poor	.18	1.46 [.84, 2.53]
Physical environment (Home):		
Difficulty getting around physical home environment	*<.001	3.51 [1.98, 6.24]
Physical environment (Community):		
Difficulty getting around local community	*.002	3.57 [1.6, 7.96]
Social environment (Friends):		
No friends outside home	.157	1.48 [.86, 2.55]
Support for activities of daily living:		
Support needed for both ADL and IADL	.831	1.12 [.41, 3.02]

*Note:* ADL = activities of daily living; ID = intellectual disability; IADL = instrumental activities of daily living.

*Indicates significance, *p* < .05.

**Table 5:** Independent Samples *T-Test and one way Between Groups ANOVA for Leisure Activities*

Leisure	Participants n = 699	%	T or f	df	Mean = 26.6	*P*
Sex:			−.522	697		.602
Female	392	56.1			26.4	
Male	307	43.9			26.93	
Age:			12.09	2		*<.001
40–49 years	198	28.3			28.87	
50–64 years	353	50.5			27.3	
65 + years	148	21.2			22.05	
Self-rated physical health (n = 11 missing):			−7.22	686		*<.001
Good	580	83			28.19	
Poor	108	15.5			18.4	
Self-rated mental health (n = 20 missing):			−4.07	677		*<.001
Good	502	71.8			28.09	
Poor	177	25.3			23.37	
Level of ID (n = 54 missing):			42.45	2		*<.001
Mild	153	21.9			32.92	
Moderate	300	42.9			27.18	
Severe/Profound	192	27.5			20.5	
Physical environment (Home) (n = 5 missing):			−8.84	692		*<.001
No difficulty navigating home environment	575	82.3			28.67	
Difficulty navigating home environment	119	17			17.35	
**Leisure**	**Leisure n = 699**	**%**	**t**	**df**	**Mean**	***T*-test or ANOVA *p***
Physical environment (Community) (n = 7 missing):			−6.67	690		*<.001
No difficulty accessing local community	248	35.8			31.16	
Difficulty/does not access local community	444	64.2			24.26	
Friends outside home (n = 6 missing):			8.4	691		*<.001
Friends outside home	392	56.1			30.29	
No friends outside home	301	43.1			22.01	
Living situation			27.96	2		*<.001
Independent/Family	113	16.2			31.18	
Community group home	303	43.4			29	
Residential center	283	40.5			22.27	
Support for activities of daily living (n = 27 missing):			−6.95	670		*<.001
Support for either ADL or IADL	103	14.7			34.75	
Support for both ADL and IADL	569	81.4			25.13	
Productivity activities			−9.18	697		*<.001
Engages in productivity role	597	85.4			28.46	
Not currently engaged in productivity role	102	14.6			15.93	

*Note:* ADL = activities of daily living; ID = intellectual disability; IADL = instrumental activities of daily living.

*Indicates significance, *p* < .05.

When controlling for all other variables, difficulty getting around the home environment was the strongest predictor of preclusion from engagement in productive activities. Those who reported difficulty getting around their home environment were 3.5 times less likely (95% CI: 1.9–6.2) to be engaged in a work activity compared to those who had no difficulty getting around their home.

Those who had difficulty getting around the local community were 3.6 times (95% CI: 1.6–7.9) less likely to be engaged in a work activity than those who had no difficulty getting around the local community. Participants who reported to be in poor physical health were 1.9 times (95% CI: 1–3.4) less likely to be engaged in a productivity role than those who reported to be in good physical health.

Age was a significant predictor of engagement in a productivity role when controlling for all other variables in the regression model, with those aged 65 years and over 2.8 times (95% CI: 1.3–5.7) less likely to be engaged in a productivity role compared to those in the younger 40–49 years group. However, no significant differences were found between those in the youngest (40–49 years) and middle age (50–64 years) group.

Sex, living situation, mental health, support required for ADL and friends outside the home were not found to be significant predictors of engagement in productivity when adjusting for all other variables.

### Leisure

Only 3.2% (n = 22) of the IDS-TILDA population were not engaging in leisure activities in the past year. The majority (96.9%, n = 677) engaged in at least one leisure activity once or twice per year. Higher percentages were reported for those who reported good self-rated mental and physical health, moderate level of ID, no difficulty moving around home, engaged in a productivity role, and required support for both ADL and IADL. Independent samples *t*-tests and one-way ANOVA were completed to identify factors that significantly affect participation in leisure activities. The findings are shown in Table . Significant findings at *p* < .001 level were found for physical health, mental health, difficulty with accessing the physical environment of home and community, level of ID, type of living situation, and presence of friends outside the home. Age was also identified as a significant factor influencing engagement in leisure activities. Sex was not identified as a significant factor influencing engagement in leisure activities.

#### Multiple linear regression

Multiple linear regression analysis examined the association between leisure participation and age, sex, level of ID, living situation, physical health, mental health, physical environment (home and community), social environment, and support required for ADL.

Dummy variables were created for categorical variables that included three or more categories: age, level of ID, and living situation to reduce the risk of multicollinearity, which occurs when two independent variables are highly related in a regression model. Variance inflation factors were all <1.5, and tolerance values all <1, indicating multicollinearity was not impacting on the overall regression model.

Results of multiple linear regression are shown in Table 6. The full model containing all 11 independent variables was statistically significant, (*p* < .001) indicating that the model was able to identify predictors of engagement in leisure activities. The R square value indicated that the model explained 31.6% of variance in leisure engagement.

**Table 6:** Multiple Linear Regression for Leisure

Leisure	Unstandardized coefficient (B)	*P*	Beta 95% confidence intervals
Sex:			
Female	−.83	.375	−.031 [−2.67, 1.01]
Age:			
50–64 years	−1.07	.322	−.041 [−3.19, 1.05]
65 + years	−3.04	*.026	−.093 [−5.7, −.37]
Level of ID:			
Mild	2.9	*.020	.091 [.45, 5.34]
Severe/profound	−4	*<.001	−.139 [−6.22, −1.79]
Living Situation			
Independent/family	−3	*.043	−.079 [−5.9, −.1]
Residential center	−2.45	*.018	−.091 [−4.47, −.43]
Physical health:			
Poor physical health	−3.42	*.012	−.094 [−6.1, −.74]
Mental health			
Poor mental health	−2.06	.061	−.068 [−4.2, .09]
Physical environment (Home)			
Difficulty	−4.68	*<.001	−.135 [−7.28, −2.08]
Physical environment (Community)			
Difficulty	−1.5	.170	−.053 [−3.64, .65]
Social environment (Friends)			
No friends outside home	−4.35	*<.001	−.164 [−6.31, −2.38]
Support for activities of daily living			
Support required for both ADL and IADL	−3.27	*.021	−.088 [−6.04, −.49]
Productivity			
Not currently engaged in productivity role	−7.16	*<.001	−.192 [−9.91, −4.4]

*Note:* ADL = activities of daily living; ID = intellectual disability; IADL = instrumental activities of daily living.

*Indicates significance, *p* < .05.

The strongest predictor of preclusion from engagement in leisure activities was not being engaged in a productivity role when adjusting for all other variables. Those who were not currently engaged in a productivity role were 7.2 times less likely to be engaged in leisure activities compared to those who were engaged in a productivity role.

Participants who reported difficulty getting around their home environment were 4.7 times less likely to engage in leisure activities compared to those with no difficulty getting around their home. This was closely followed by those who reported that they did not have friends outside the home, who were 4.4 times less likely to engage in leisure activities than those who did have friends outside the home in the multiple regression.

Participants who had severe/profound level of ID were 4 times less likely than those with moderate level of ID to engage in leisure activities, and those with mild ID were also 2.9 times more likely to engage in leisure activities than those with moderate level of ID.

Participants who were aged 65 years and older were 3 times less likely than those aged 40–49 years to be engaged in leisure activities. There was no significant difference between the youngest (40–49 years) and middle age (50–64 years) groups.

Participants who reported poor physical health were 3.4 times less likely than those in good physical health to engage in leisure activities when controlling for all other variables.

Those who required support for *both* ADLs and instrumental activities of daily living (IADL) were 3.3 times less likely than those who required support for *either* ADLs or IADLs to be engaged in leisure activities.

When controlling for all other variables in the model, living situation was also a significant predictor of preclusion from leisure engagement, with those in residential centers 2.5 times less likely than those in community group homes to engage in leisure activities. Those living independently or with family were also 3 times less likely to engage in leisure activities than those living in community group homes.

Sex, level of difficulty getting around community environment, and mental health were not found to be significant predictors of leisure engagement when adjusting for other variables in the model.

## Discussion

This study aimed to add to the understanding of the occupational needs of adults aging with ID, by examining factors that may preclude engagement in work and leisure activities. The sample was drawn from a nationally representative sample, and attempted to examine the influence of selected objective factors within the person and environment that influence participation in work and leisure activities.

The findings demonstrated that a variety of factors influence engagement in productivity and leisure activities. Overall, difficulty getting around home and community environment, poor physical health, and older age (65 + years) were the strongest predictors of preclusion from engagement in productivity and leisure activities. Other factors found to be significant for preclusion from engagement in leisure activities only, included: living in a residential center, level of ID, not being engaged in a productivity role, no friends outside the home, support required for both ADLs and IADLs, and independent/family living situation.

### Moving Around Home and Community

The present study showed that an appropriate and accessible home environment is important in supporting participation in work and leisure occupations for adults aging with ID. This study found that when a person has difficulty navigating their home environment, it negatively affects their opportunities to engage in important productivity and leisure activities outside of the home as well.

Previous qualitative studies have examined the characteristics of the home environment in supporting or hindering participation in occupation for adults with intellectual disabilities living in community group homes, or living independently. These studies have identified barriers within the physical home environment can hinder participation in ADL, and community participation (Ashley et al., 2019; Kåhlin et al., 2016).

Blaskowitz et al. (2021) and Washington et al. (2021) also highlighted the role of occupational therapy in the creation of environments that support participation for adults with ID. The present study extends the work of previous research, as it includes a broader range of living situations, including residential centers, community group homes, and people living independently, or with family. Using a quantitative approach, it offered a broader perspective of participation in work and leisure occupations for older adults aging with ID in Ireland, and adds to the evidence base, along with existing valuable qualitative studies that examine participation within a specific context (e.g., community group homes).

#### Aging in place

The findings of this study showed it is particularly relevant for occupational therapists to identify and address barriers and opportunities in both home and community environments which can improve engagement in productive and leisure activities. Occupational therapists can work with older adults with intellectual disabilities, and their support networks, to support the creation of accessible physical home environments, and universal design principles that facilitate participation in daily life.

In recent years, increased attention has been given to models that support a person to continue living in their preferred living environment, often known as “aging in place.” Washington et al. (2021) concluded that occupational therapists have an important role in supporting adults aging with ID to age in place. In Ireland, this is reflected in policies such as the National Positive Aging Strategy (Department of Health, 2013).

These findings are particularly relevant at present, given that the current Irish disability policy focuses on transitioning from day services, and moving toward individualized community-based day activation services (Health Service Executive, 2012). It is possible that this could result in older adults with ID spending increased amounts of time in their home environment, rather than attending traditional group-based services during the day, which further emphasizes the importance of an appropriate home environment (Kåhlin et al., 2016).

As well as addressing the accessibility of the physical environment, there are opportunities for occupational therapists to support adults aging with ID in other domains of life (Blaskowitz et al., 2021). Descriptive statistics in this study showed many people aging with ID don't have friends outside of their home, and require support for ADL. Washington et al. (2021) reported there are opportunities for occupational therapists to work with adults aging with ID to focus on developing friendships in and outside the home, and supporting participation within the community. Leisure activities are a valuable way to maintain and expand social networks through participation in occupation within communities.

### Physical Health

Occupational therapists can work as part of a multidisciplinary team to assist in preventing or reducing the impact of physical health needs on participation in daily life (including work and leisure occupations) as people with ID grow older. Poor physical health was identified as a factor that precluded participation in work and leisure activities for adults aging with ID in Ireland in this study. The current study found that 15.7% (n = 108) of respondents reported poor physical health. Previous research indicates that adults aging with ID experience a range of age-related conditions earlier than adults in the general population (Bershadsky et al., 2012; Fesko et al., 2012). For occupational therapists, participation in occupation is intrinsically related to health, and this highlights the potential for occupational therapists to collaborate with multidisciplinary colleagues and adults aging with ID to develop and implement occupation-focused health promotion approaches to enable adults with ID to maintain their health and sustain participation in daily life as they grow older.

### Aging and Retirement

Adults aged 65 years and older were significantly less likely to be engaged in productivity and leisure activities than those who were younger (aged 40–49 years) in this study. Previous research has found that people aging with ID wish to maintain or even increase their participation in meaningful occupations as they grow older (Fesko et al., 2012). Given that the present study found decreased rates of participation in work and leisure activities as adults with ID grow older, this indicates that adults aging with ID may not be afforded the opportunities to participate in work and leisure activities that they may wish, consistent with findings in other studies (e.g., Bigby et al., 2011; Charnley et al., 2019).

These findings indicate the need for occupational therapists to collaborate with adults aging with ID, and their support networks to sustain and enhance participation in work and leisure activities as adults with ID grow older. This could include working at an individual level with adults aging with ID and their support staff to analyze activities and occupations that best match their abilities and interests, and providing specialized advice to support their participation in meaningful work and leisure activities. Occupational therapists can play a significant role in advocating for and implementing occupation-focused approaches that enable adults with ID to continue to participate in meaningful work and leisure opportunities as they grow older (Blaskowitz et al., 2021; Johnson et al., 2019). Occupational therapists can work directly with adults aging with ID, or through consultative or coaching approaches with support staff or family members to facilitate participation in work or leisure occupations (Johnson et al., 2019). Furthermore, occupational therapists can play an important role in education, and advocating for the importance of occupation, and the associated benefits for health and well-being at a broader level within disability and healthcare services (Johnson et al., 2019).

## Limitations

Logistic and linear regression techniques were useful in identifying predictors of and barriers to engagement in productivity and leisure activities of people aging with ID. Such findings cannot imply causation, as they are based on one wave of data. The authors were able to access and analyze data from wave 2 of IDS-TILDA, gathered in 2014. Future research would benefit from the examination of patterns of engagement in daily activities over time.

This study attempted to examine predictors of engagement in productivity and leisure occupations for adults aging with ID using an occupational therapy perspective. This study took a quantitative approach and gathered data that was objective in nature. This was important so that a broad range of adults aging with ID could participate in the study, including self-reporting respondents, and proxy respondents. There were limitations to this approach. Information on the personal meaning of productivity or leisure occupation, or the influence of more subtle spiritual, institutional, or cultural factors could not be gathered, due to the subjective nature of such data. Future studies would benefit from gathering additional data in relation to the importance or relevance of the productivity or leisure occupation for the person, as well as examining additional factors such as spirituality, cultural and institutional factors that influence participation in work and leisure occupations.

In this study, a variety of activities related to productivity was considered, for example, employment, training, education, day services, and sheltered employment. These activities were examined as a group of productivity activities, and weren't differentiated during analysis. Future studies could explore how meaningful occupational engagement is facilitated within different productivity occupations to explore how adults aging with ID experience occupation in these contexts.

## Conclusions

Factors within the physical environment of the home, physical health, and older age were identified as the main barriers to engagement in work and leisure activities for people aging with ID. This study found that people with ID participated less in work and leisure occupations as they age, and poor physical health was an important predictor of preclusion from engagement in work and leisure. Occupational therapists have opportunities to develop and implement approaches to maintain health and well-being through meaningful work and leisure occupations.

This study demonstrates the importance of an appropriate and accessible physical home environment in supporting participation in work and leisure activities, both within the home, and in the local community. It points to important areas of practice for occupational therapists using universal design principles, and provision of practical supports and advice to adults aging with ID, families, and service providers in supporting adults aging with ID to “age in place.”

In Ireland, there is a need to work with key stakeholders within housing authorities and management of disability services to promote universal design, and aging in place for adults with ID as they grow older.

## Key Messages

- The physical environment plays a significant role in facilitating or hindering participation in work and leisure activities for adults aging with ID in Ireland.
- Adults aging with ID who reported poor physical health, or were aged over 65 years participated less in work and leisure occupations.
- Occupational therapists can work with adults aging with ID, and their support networks to foster participation in work and leisure occupations as they grow older.
